# Migration: A risk factor for psychosis?

**DOI:** 10.1192/j.eurpsy.2021.1944

**Published:** 2021-08-13

**Authors:** E. Spaho, V. Alikaj, E. Dashi, V. Skendi

**Affiliations:** 1 Department Of Neuroscience, University Hospital Center “Mother Theresa”, Tirane, Albania; 2 Faculty Of Medicine, Tirana Medical University Albania, Tirane, Albania

**Keywords:** Risk factors, psychosis, schizophrénia, Migration

## Abstract

**Introduction:**

Emigration is a complex process of social changing through which an individual moves from a certain cultural environment/context to another, aiming to achieve persistent or long-term residency, causing distress. There is sustainable evidence that incidence of all forms of psychosis is higher in migrants.

**Objectives:**

This study aims to gather data of other research conducted in the field according to emigration as a risk factor for development of different psychosis.

**Methods:**

Scientific articles searched in MEDLINE, regarding the incidence of mental disorders in different emigrant populations, for the period 1995 - 2015.

**Results:**

The average relative risk of schizophrenia and of other psychosis occurrence among first generation emigrants was 2.7 (95% confidence interval [CI]=2.3-3.2). Statistical analysis performed among studies of first and second generation of emigrants, and among studies which don’t make difference between generations, results in a relative risk of 2.9 (95% CI=2.5-3.4) of mental illness.

**Conclusions:**

The data presented in this study emphasize the impact of migration on central symptoms of schizophrenia. Emigration process, cultural and social adaptation, play an important role on the individual mental health.

**Disclosure:**

No significant relationships.

